# Determining the Swelling Behavior and Tensile Strengths of Commercially Produced Buna‑N O‑Rings and Stereolithographic Additively Manufactured O‑Rings after Exposure to Mixtures Containing Jet Fuels, Synthetic Fuels, and Fuel Surrogate

**DOI:** 10.1021/acsomega.5c04803

**Published:** 2025-07-24

**Authors:** Dianne J. Luning Prak, David Graham, Kara Hunt, Micah Evans, Terrence Dickerson, Jonathan Slager, Jim S. Cowart

**Affiliations:** † 32722 Chemistry Department U.S. Naval Academy, 572M Holloway Road, Annapolis, Maryland 21402, United States; ‡ Naval Air Warfare Center Aircraft Division (NAWCAD) Air Systems Group; Building 2360, NAS Patuxent River, Annapolis, Maryland 20670, United States; § Mechanical and Nuclear Engineering Department U.S. Naval Academy, 590 Holloway Road, Annapolis, Maryland, 21402, United States

## Abstract

Synthetic fuels are among the portfolio of fuels that enable operational energy resilience. This work investigates the physical properties of mixtures of military jet propellant fuel JP-5 with synthetic paraffinic kerosene (alcohol-to-jet, ATJ-SPK), formulates a surrogate mixture for ATJ-SPK, and explores the swelling behavior and tensile strengths of commercially manufactured Buna-N O-rings and stereolithographic additively manufactured (SLA) acrylate O-rings after exposure to fuels, surrogate, and fuel mixtures with additives. An ATJ-SPK surrogate was formulated (0.185 mole fraction of *iso*-cetane in *iso*-dodecane isomers) whose density, speed of sound, viscosity, flash point, and swelling matched those of ATJ-SPK. ATJ-SPK and its surrogate swelled Buna-N (∼3%) and SLA (∼5%) O-rings less than did JP-5 (∼23% Buna-N, ∼18% SLA). Adding cyclic and aromatic compounds to mixtures of JP-5 with ATJ-SPK or its surrogate increased swelling and decreased the tensile strength of the O-ring. SLA O-rings had tensile strengths lower than those of Buna-N O-rings. The densities of some mixtures met military fuel specifications, while many flash points were too low. This work shows that SLA O-rings can produce adequate swelling behavior and that cyclic and aromatic compounds can be used to enhance swelling, but the fuel’s physical properties must be examined to determine if they are adversely impacted.

## Graphical abstract

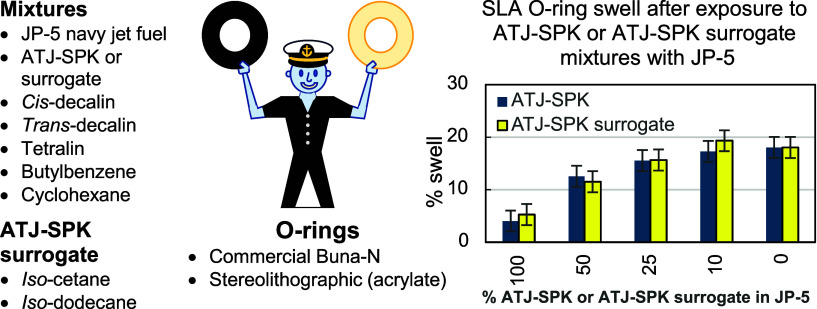

## 1 Introduction

The production and use of synthetic fuels expand the portfolio of potential energy sources for the transportation sector. In the commercial aviation sector, synthetic aviation fuels (SAFs) have been approved for use in mixtures with petroleum-based commercial aviation fuel up to 50%.^1^ The formulations of synthetic fuels, whose composition may differ from petroleum-based fuels,^1^ can affect the physical properties of fuels as well as their interactions with engine components such as O-rings. Many SAFs contain mostly linear and branched hydrocarbons but lack aromatic and cyclic components, which also leads to less O-ring swelling than petroleum-based fuels and changes the sealing ability of the associated fuel system O-rings.^2–5^ Researchers have explored adding various aromatic and cyclic compounds to a subset of SAFs as a way to enhance the swelling.^6,7^ The goal of this study was to explore the swelling and tensile strength of commercial and stereolithographic additively manufactured O-rings after exposure to synthetic aviation fuel (alcohol-to-jet fuel derived from iso-butanol, ATJ-SPK), military jet fuel JP-5, and mixtures. Due to JP-5’s higher flash point requirement that is necessary for safe shipboard operation, JP-5 differs from commercial aviation fuel, so materials testing with JP-5 is important for understanding the impacts on military systems. Under certain circumstances, the navy uses jet fuel in its diesel engines, so it is important to understand how fuel mixtures affect diesel engine components and fuel systems. This work also designed a surrogate mixture to mimic the properties of the synthetic fuel to explore the swelling behavior of the tested O-rings.

The compression of the O-rings between mechanical parts helps prevent the leakage of gases and liquids. The swelling of some O-rings in the presence of organic liquids enhances their sealing ability, but a high swelling may result in O-ring damage. Buna-N O-rings have been found to swell in the presence of fuels and are used in military applications. The swelling behaviors of Buna-N polymer materials (rectangular samples,^4,8^ cylindrical slices of O-rings,^2,4^ and whole O-rings^6,9,10^ ) in synthetic fuels, pure components, and their mixtures have been reported in the literature. Many of the synthetic fuels lack aromatic content: Fischer–Tropsch fuel (FT-SPK, less than 1% aromatic compounds), alcohol-to-jet fuel (Gevo ATJ-SPK from iso-butanol, mostly branched components), Sasol isoparaffinic kerosene (IPK, 96.9% branched hydrocarbons, 2.3% cyclic, 0.4% branched, and 0.3% aromatic), and synthetic isoparaffinic fuel (SIP, comprised of mostly farnesane).^2,7,9^ Exposures of Buna-N to FT-SPK,^9^ ATJ-SPK,^2^ IPK,^7^ and SIP^6^ have been reported to produce volume changes of 0.7%, 0.5%, 0.05%, 0.5%, and −1.37%, respectively. These swells are much less than the 16.2% reported after contact with JP-5.^9^ Researchers have explored using additives containing alcohol, aromatic, and cyclic functional groups to increase swelling. Mixing the FT-SPK with various aromatic compounds produced Buna-N O-ring volume increases of 6% to 13%,^11^ while doping this fuel with 1 wt % benzyl alcohol caused the O-rings to swell to the same level as the jet fuel.^9^ Fu and Turn^6^ reported up to 24% swells from exposure to 25% aromatic mixtures in SIP. The upper limit for aromatic compounds in military jet fuel JP-5 is 25 vol %.^12^ The largest swelling of Buna-N nitrile O-rings after contact with Sasol IPK doped with 8% by volume of alkenes, aromatic, and cyclic compounds was found to be 3.1% for a mixture of IPK with 8% ethylbenzene.^7^ For ATJ-SPK doped with 8% of various compounds, the biggest volume change was 17.4% for a mixture of ATJ-SPK with 8% biphenyl.^2^ No study, however, has explored the impact of mixing ATJ-SPK with JP-5 on O-ring swelling.

O-rings can also be produced using additive manufacturing (AM) technologies. AM enables the rapid production of small quantities of customizable parts and is adaptable to emergency situations. Stereolithography (SLA) can produce soft polymers by selectively focusing ultraviolet light (UV) into a vat containing UV photocurable liquid monomers and selectively photopolymerizing the material into a specific shape following a pattern from a computer-aided design (CAD) model.^13^ The parts formed by this process will have mechanical properties and swelling behaviors that differ from traditional commercially produced parts.^14–17^ The surfaces will differ when exterior supports are removed from the AM parts (e.g., indentations), and the interior may differ if unreacted monomer remains.^17–20^ Conventional molding processes may also produce surface defects, but standards, such as those promulgated by the Society for Automotive Engineers,^21^ specify the extent of allowed surface defects. An advantage of using SLA O-rings is that they can be rapidly produced on demand (typically in small quantities) when needed in emergency situations.

Researchers will often prepare mixtures of known compositions (surrogate mixtures) to match those of the fuel and then use those mixtures to explore the combustion or other properties of the fuels. Surrogates with few components are more easily modeled, while those with more components readily capture more properties of interest. Previously used metrics for surrogate formulation have included density, speed of sound, viscosity, derived cetane number, octane number, flash point, total sooting index, lower heating value, thermal conductivity, H/C molar ratio, and volatility measurements through a distillation curve.^22–39^ The properties (density, bulk modulus, viscosity, surface tension, and flash point) of a previously studied ATJ-SPK fuel were best matched using mixtures with mass fractions ranging from 0.2001 to 0.5000 of 2,2,4,4,6,8,8-heptamethylnonane in 2,2,4,6,6-pentamethylheptane (one isomer of *iso*-dodecane),^40^ while an optimal surrogate of 0.25 mass fraction of *iso*-cetane in *iso*-dodecane isomers successfully matched ATJ-SPK behavior in combustion experiments with JP-5.^41^ These studies show that these branched components are good candidates for use in developing a surrogate for the currently tested ATJ-SPK fuel.

Most of the work on O-ring swelling of the ring has focused on commercial aviation fuel with less work emphasizing military jet fuel, JP-5. The specifications for JP-5 differ from those of commercial aviation, with differences in density ranges and minimum flash point (60 °C for JP-5 and 38 °C for commercial aviation fuel).^12,42^ The goals of this study were to (1) explore the effect of mixing ATJ-SPK with JP-5 on fuel physical properties, (II) formulate a surrogate for ATJ-SPK, and (III) determine the impact of mixtures of JP-5 with ATJ-SPK and its surrogate with and without dopants on the swelling and tensile strength behavior of commercial Buna-N and SLA O-rings. The approach taken in this study is shown in Figure 1.

**Figure 1:**
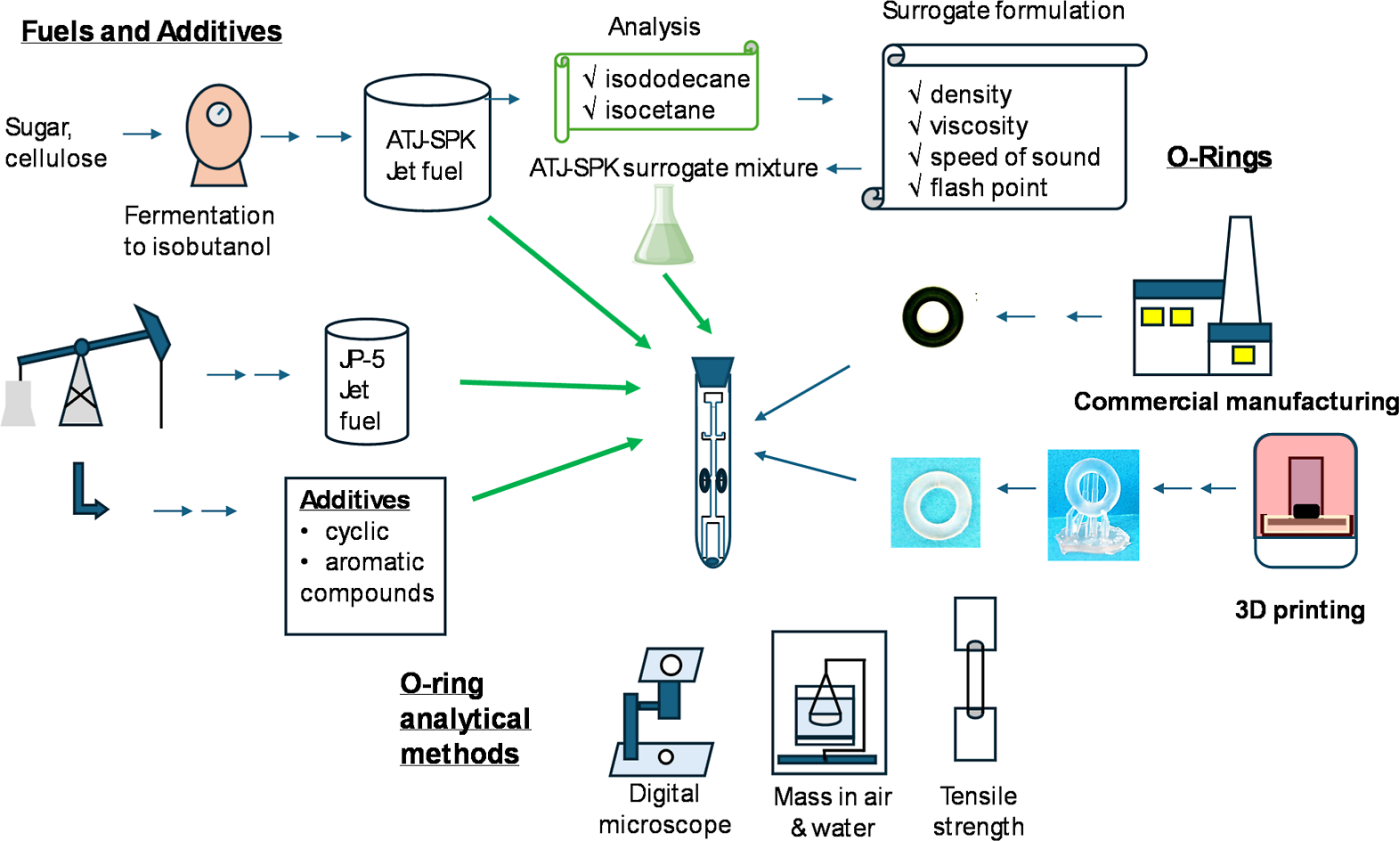
Pictorial representation of exploring the swelling and tensile strength of AM and commercial O-ring after exposure to synthetic jet fuel (ATJ-SPK), a surrogate ATJ-SPK mixture, military jet fuel JP-5, and mixtures with additives.

## 2 Experimental and/or Computational Methods

### 2.1 Materials

The organic materials used in this study were 2,2,4,4,6,8,8-heptamethylnonane (*iso*-cetane, Acros, 99% pure Acros), *iso*-dodecane isomers (bottle purity: 80%; measured purity 98% of two isomers, see Supporting Information), *trans*-decalin (TCI, purity 98% pure), *cis*-decalin (TCI, 98% pure), butylbenzene (TCI, 99% pure), cyclohexane (Alfa Aesar, 99.5%), and 1,2,3,4-tetrahydronaphthalene (tetralin, Sigma-Aldrich, 99% pure). These compounds were chosen to include aromatics and cyclic compounds that are known to swell Buna-N. The alcohol-to-jet fuel (ATJ-SPK) and JP-5 jet fuel were provided by the Naval Air Warfare Center Aircraft Division (NAWCAD) fuel’s group. The chemical compositions of the JP-5 and ATJ-SPK were determined using a GC × GC-FID test method developed by the Department of Defense (Test Method 7508.0 Method for Detailed Hydrocarbon Analysis of Middle Distillate Fuels by Two-Dimensional Gas Chromatography).^43^ JP-5 contained 19.4% linear alkanes, 29.3% iso-alkanes, 35.6% cycloalkanes, 10.2% aromatic compounds, and 5.4% cycloaromatic compounds. The ATJ-SPK contained only iso-alkanes with mostly 12 and 16 carbon atoms.

Commercially produced O-rings and SLA O-rings were used in this investigation. The commercial O-rings were a Ford Motorcraft O-ring (CM-4717) and two Buna-N O-rings [AS (AS3578-203) and MS (MS29513-203)] purchased from McMaster Carr. The SLA O-rings were printed with Flex 80A liquid polymer resin (Formlabs, Somerville, MA) using a Formlabs Form 3 Low Force Stereolithography printer with an 85 μm laser spot size, a 0.100 μm layer height, and 25 μm XY resolution. The parts were orientated at approximately 15° from vertical with a full raft and support only touching the outer edges of the O-ring (Figure 2). All O-rings were postprocessed by washing for 20 min in a Formlabs Form Wash agitated wash system with isopropanol, curing in a Formlabs Form Cure L oven with UV light for 10 min at 60 °C, and manually removing all support material.

**Figure 2:**
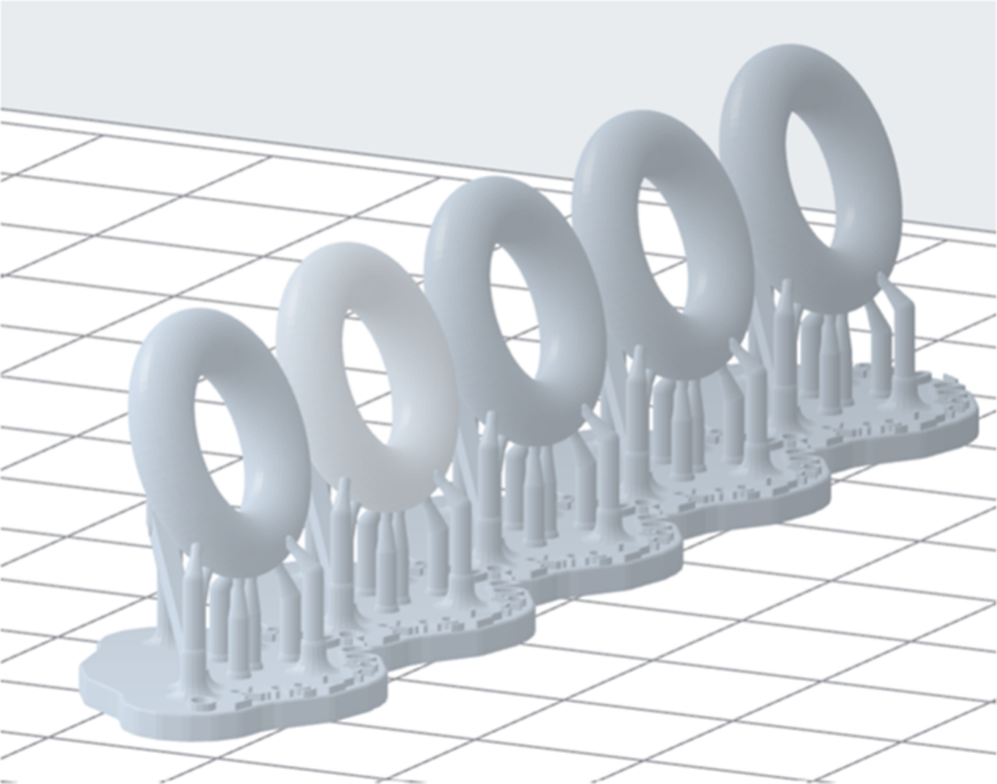
Print orientation of SLA O-rings.

### 2.2 Physical Properties and Formulation of Surrogate Mixtures for ATJ-SPK Fuel

The approach used to formulate a surrogate mixture for ATJ-SPK was to match as closely as possible the density, viscosity, speed of sound, and flash point of the mixtures with those properties of ATJ-SPK. The density and speed of sound were measured using an Anton Paar DSA 5000 density and velocity of sound analyzer, and the viscosity was measured using an Anton Parr SVM 3000 viscometer. The flash point was measured using a SETAflash series 8 flash point tester. The surrogate mixtures were made by varying the proportions of *iso*-cetane and *iso*-dodecane isomers. These components had been used in previous studies for developing a surrogate for earlier versions of this type of fuel.^41^

### 2.3 O-Ring Swelling and Tensile Test Measurements

For each liquid mixture tested, three O-rings were submerged for 7 days using previously reported devices^15,16^ at room temperature, 21 °C (see Figure 1). Volume changes were determined from photographed images of the O-rings and from mass measurements before and after exposure to the fuels or fuel mixtures. Digital photos of the O-rings were taken before and after exposure using an Opti-Tek Scope and analyzed using a Python program^15^ to find their inner (*R*_inner_) and outer (*R*_outer_) radii. O-rings are the shape of a torus, and their volume (*V*) can be determined using eq 1.

1 $$V=025\pi^{2}\times {\left(R_{outer}-R_{inner}\right)}^{2}\times \left(R_{outer}+R_{inner}\right)$$

O-ring masses were measured in air and ultrapure water using a Mettler Toledo Density Kit before and after exposure to the fuel mixture. Equation 2 shows that the volume change can be determined from the masses in air (*W*_1_) and water (*W*_2_) before exposure and in air (*W*_3_) and water (*W*_4_) after exposure.

2 $$volume\;change=\frac{\left(W_{2}+W_{3}\right)-\left(W_{1}+W_{4}\right)}{W_{1}-W_{2}}\times 100$$

Both methods can be affected by evaporation in air during measurement, which can produce an underestimation of volume change, while the mass method is also affected by any dissolution in the water phase, which could produce an overestimation of volume change.

The tensile strength of each O-ring was measured using an Admet MTEST Quattro tensile tester equipped with half-shell adapters for testing O-rings of this small size (see Luning Prak et al.^16^). The adapters were lubricated with mineral oil prior, the O-ring placed around them, and the half shells were pulled apart at a rate of 500 mm/min, which is the speed specified in ASTM D1414.28. This tensile tester recorded the force reading at 90% of the maximum, corresponding to when the O-ring broke due to the applied force. The tensile strength (MPa) was calculated by dividing the force (N) by the cross-sectional area of the O-ring (mm^2^). The area was calculated from the dimensions of the O-ring determined from the digital images immediately before the tensile test was conducted.

## 3 Results and Discussion

### 3.1 Physical Properties of Fuel Mixtures and Optimal Fuel Surrogate

The optimal surrogate mixture was determined to contain 0.185 mole fraction of *iso*-cetane in *iso*-dodecane isomers (0.232 mass fraction). The percentage differences between the surrogate’s and ATJ-SPK’s properties were 4%, 0.04%, 0.03%,and 0.2% for viscosity, density, speed of sound, and flash point, respectively (Table 1). This 0.232 mass fraction of *iso*-cetane in *iso*-dodecane isomers is consistent with 0.25 mass fraction found in a surrogate developed for an earlier version of this fuel,^41^ and within the mass fraction range of 0.2001 to 0.5000 2,2,4,4,6,8,8-heptamethylnonane in 2,2,4,6,6-pentamethylheptane given in another study.^40^

**Table 1:** Physical Properties of Fuels and Mixtures

fuel and mixtures	viscosity at 20 °C (mm^2^/s)	density at 15 °C (kg/m^3^)	speed of sound at 20 °C (m/s)	flash point (°C)
ATJ-SPK	2.06	759.4	1224.9	50.3
ATJ-SPK surrogate	2.15	759.1	1225.3	50.2
90% ATJ-SPK + 10% *cis*-decalin	2.18	774.6	1250	52
90% ATJ-SPK_surrogate_ + 10% *cis*-decalin	2.25	773.7	1249	52
75% ATJ-SPK + 25% *cis*-decalin	2.37	**796.8**	1286	53
75% ATJ-SPK_surrogate_ + 25% *cis*-decalin	2.43	**796.0**	1286	53
50% ATJ-SPK + 50% *cis*-decalin	2.74	**833.8**	1346	56
50% ATJ-SPK_surrogate_ + 50% *cis*-decalin	2.80	**832.5**	1344	55
50% ATJ-SPK_surrogate_ + 50% tetralin	2.02	**867.5**	1359	59
50% ATJ-SPK + 50% JP-5	2.00	783.0	1275	56
50% ATJ-SPK_surrogate_ + 50% JP-5	2.03	782.9	1275	56
45% ATJ-SPK + 45% JP-5 + 10% *cis*-decalin	2.12	**795.5**	1293	57
45% ATJ-SPK_surrogate_ + 45% JP-5 + 10% *cis*-decalin	2.14	**795.0**	1293	57
45% ATJ-SPK, 45% JP-5, 5% *cis*-decalin, 5% butylbenzene	1.96	**793.8**	1288	56
45% ATJ-SPK_surrogate_,45% JP-5, 5% *cis*-decalin, 5% butylbenzene	1.98	**792.8**	1287	56
45% ATJ-SPK + 45% JP-5 + 10% butylbenzene	1.82	**790.6**	1281	56
45% ATJ-SPK_surrogate_ + 45% JP-5 + 10% butylbenzene	1.84	**790.9**	1282	56
45% ATJ-SPK + 45% JP-5 + 10% tetralin	1.96	**802.2**	1295	57
45% ATJ-SPK_surrogate_ + 45% JP-5 + 10% tetralin	1.99	**802.4**	1296	57
25% ATJ-SPK + 75% JP-5	2.00	794.6	1298	59
25% ATJ-SPK_surrogate_ + 75% JP-5	2.01	794.3	1298	**60**
20% ATJ-SPK + 70% JP-5 + 10% *cis*-decalin	2.09	**804.7**	1313	**60**
20% ATJ-SPK_surrogate_ + 70% JP-5 + 10% *cis*-decalin	2.11	**805.3**	1314	**60**
20% ATJ-SPK_surrogate_ + 70% JP-5 + 10% tetralin	1.98	**812.6**	1316	**60**
20% ATJ-SPK + 70% JP-5 + 10% *trans*-decalin	2.01	**802.1**	1307	58
20% ATJ-SPK + 70% JP-5 + 10% cyclohexane	1.85	**793.2**	1294	NM
10% ATJ-SPK + 90% JP-5	2.00	**801.4**	1312	**62**
10% ATJ-SPK_surrogate_ + 90% JP-5	2.00	**801.0**	1311	**62**
5% ATJ-SPK + 85% JP-5 + 10% *cis*-decalin	2.09	**810.9**	1325	**61**
5% ATJ-SPK + 85% JP-5 + 10% *trans*-decalin	2.01	**808.2**	1329	**60**
5% ATJ-SPK + 85% JP-5 + 10% cyclohexane	1.86	**799.6**	1306	<21
JP-5	2.01	**805.7**	1320	**65**

Some of the physical properties of ATJ-SPK fuel are similar to those of JP-5 jet fuel (Table 1). The viscosity is similar, but the speed of sound, density, and flash point are lower than the values for JP-5. The range of densities allowed for military jet fuel is 788 to 845 kg·m^3^.^12^ Mixtures of ATJ-SPK or ATJ-SPK/JP-5 with some of the dopants fit within this specification and are bolded in Table 1.^12^ Viscosities are increased by adding *cis*-decalin and decreased by adding cyclohexane and butylbenzene, and very small changes occur when adding *trans*-decalin. The flash point specification for JP-5 is 60 °C minimum.^12^ Only the mixtures containing low amounts of ATJ-SPK meet this value (bold values in Table 1). It is important to note that the ASTM specification for aviation turbine fuel differs from that of military jet fuel.^42^ For example, its density range (775 to 840 kg·m^3^) and flash point (38 °C) are lower.^42^ A comparison of the properties of mixtures made with ATJ-SPK surrogate and those made with ATJ-SPK shows that the properties are similar, with the greatest differences being 1.0 kg/m^3^, 0.07 mm^2^/s, 2 m/s, and 1 °C for density, viscosity, speed of sound, and flash point, respectively.

A comparison of density and flash point can be made between mixtures of ATJ-SPK with JP-5 in the current study with those of ATJ-SPK with Jet A in an earlier study.^44^ In both studies, the ATJ-SPK densities (759.6 kg/m^3^ versus 759.4 kg/m^3^) and the jet fuel densities (both 805.7 kg/m^3^)^44^ were similar at 15 °C. Their mixtures at 10 vol % ATJ-SPK and 50 vol % ATJ-SPK had densities that were within 0.1 kg/m^3^ of each other. The flash points, however, were different. The ATJ-SPK flash points were within 2 °C of each other (48.5 °C versus 50.3 °C), while the flash points of Jet-A (43 °C)^44^ was much lower than that of JP-5 (65 °C).^44^ The mixtures of Jet A and ATJ-SPK all flashed between 42.0° and 48.5 °C^44^, while the current mixtures flashed between 50.3° and 65.0 °C.

### 3.2 Swelling and Tensile Strength of SLA and Commercial O-Rings

The O-ring swelling was determined from the difference between the volume before and after exposure determined by optical images (eq 1) and by mass measurements (eq 2.). Figures 3 and 4 show examples of optical images before and after exposure to a mixture of 10 vol % tetralin, 20 vol % ATJ-SPK surrogate, and 70 vol % JP-5. In general, the two methods produce similar values for the SLA O-rings, but the mass measurements produce slightly higher values for the Buna-N O-rings (Figure 5). Figure 6 shows swelling behavior based on image measurements taken at intermediate times before 7 days, and these data suggest that the swelling had reached its equilibrium value (see Supporting Information Figures S1–S4 for additional time series). All future discussions will use O-ring swelling based on optical images. Faulhaber et al. reported that it took from 10 to 14 days for the swelling of their Buna-N O-rings, which had been exposed to ATJ-SPK doped with 8% of various compounds, to remain nominally unchanged over at least 24 h.^2^ The difference in swell between day 7 and the end of their experiments is smaller than 2%, which is the error in the experiments reported herein.

**Figure 3:**
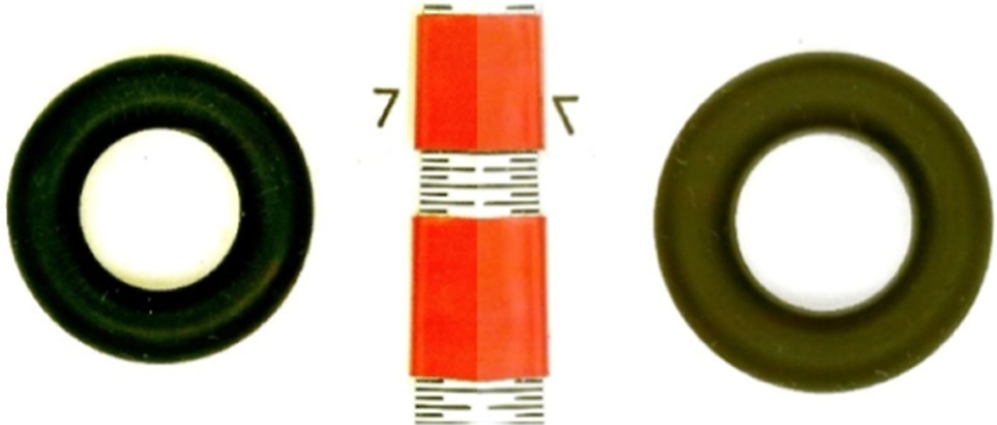
AS Buna-N O-ring before (left) and after (right) 7 day exposure to solution containing 10 vol % tetralin, 20 vol % ATJ-SPK surrogate, and 70 vol % JP-5 (35% swell). The “inch” ruler is divided into 1/64 inch segments.

**Figure 4:**
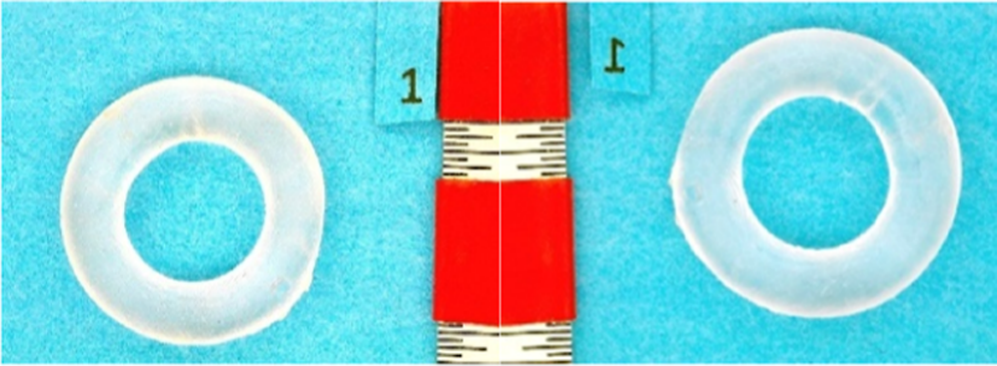
SLA O-ring before (left) and after (right) 7 day exposure to solution containing 10 vol % tetralin, 45 vol % ATJ-SPK surrogate, and 45 vol % JP-5 (21% swell). The “inch” ruler is divided into 1/64 inch segments.

**Figure 5:**
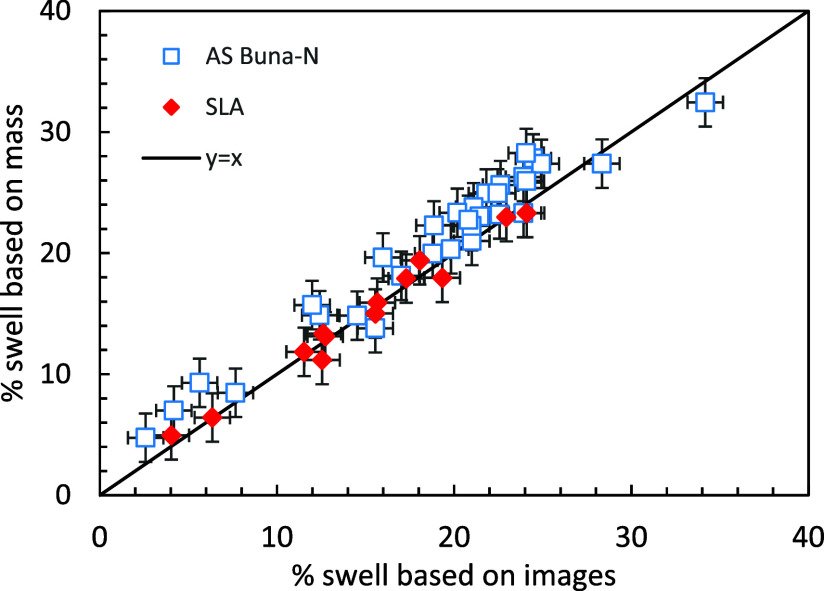
Comparison of O-ring volume changes based on mass measurements (eq 2) and images (eq 1).

**Figure 6:**
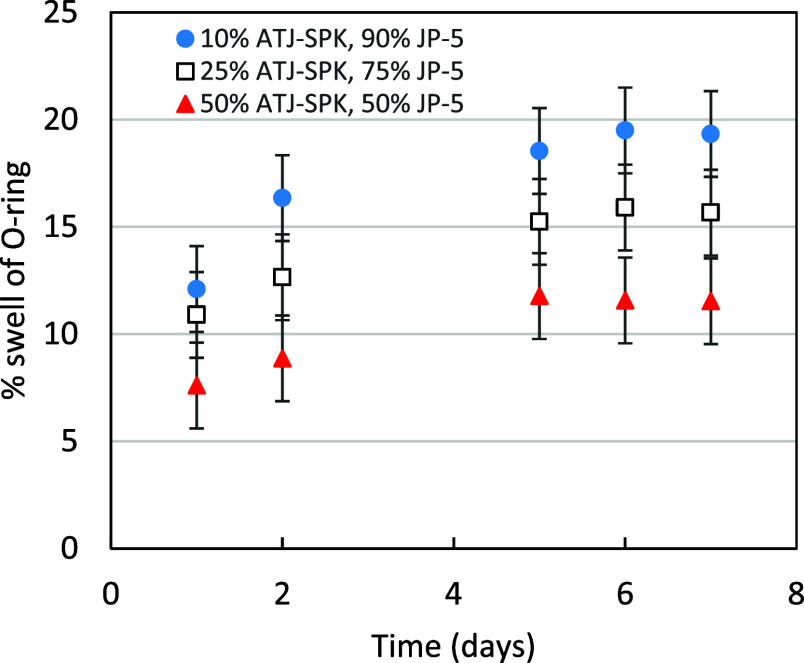
Example of time course of swelling of SLA Buna-N O-rings for mixtures of JP-5 and ATJ-SPK based on images. The mixtures were prepared as vol % ATJ in JP-5.

Initial experiments exposed the fuels to the various O-rings, and both swelling (increase in volume) and shrinkage (decrease in volume) were observed. The volume changes of the AS Buna-N, SLA, MS Buna-N, and Ford OEM O-rings after 7 day contact with ATJ-SPK were 3%, 4%, −7%, and −4%, respectively (error of ± 2%). Similar trends were found upon exposure to the ATJ-SPK surrogate with 4%, 6%, −6%, and −2% changes for AS Buna-N, SLA, MS Buna-N, and Ford O-rings, respectively. The volume swells of the AS Buna-N, SLA, MS Buna-N, and Ford O-rings after 7 days of contact with JP-5 were 22.5%, 18.1%, 2%, and −2%, respectively. These initial results suggested that AS Buna-N and SLA O-rings would be the best to explore the enhanced swelling.

One goal of these studies was to explore the swelling of mixtures of ATJ-SPK with JP-5 and determine if the addition of small amounts of cyclic or aromatic compounds could enhance Buna-N swelling to the same level as that found for JP-5. Cyclic compounds are preferable to aromatic compounds because of their clearer combustion (lower sooting). Figure 7 shows swelling behavior (% increase in O-ring volume of the O-ring above that of the unexposed O-ring) upon exposure to various fuels and fuel mixtures. Note that each mixture is designated by the volume percentage of each component in the mixture with JP-5. Mixtures containing 10 vol % ATJ-SPK in JP-5 produced swells similar to that of JP-5 (22.5 ± 2% volume swell) within the error of the measurements. Increasing the amount of ATJ-SPK in the mixtures lowered the swelling significantly. The Buna-N swellings were 18.8% and 12.4% when exposed to 25 vol % ATJ-SPK in JP-5 and 50 vol % ATJ-SPK in JP-5, respectively. To increase the swelling of these mixtures, dopants were added. For the mixtures, the cycloalkane dopants (cyclohexane, *trans*-decalin, and *cis*-decalin) increased the swelling less than the aromatic (butylbenzene) additive, which enhanced the swelling less than the cycloaromatic (tetralin) dopant. This is consistent with observations by Faulhaber et al.^2^ who found the same trends for ATJ-SPK spiked with 8 mass % of these same compounds.

**Figure 7:**
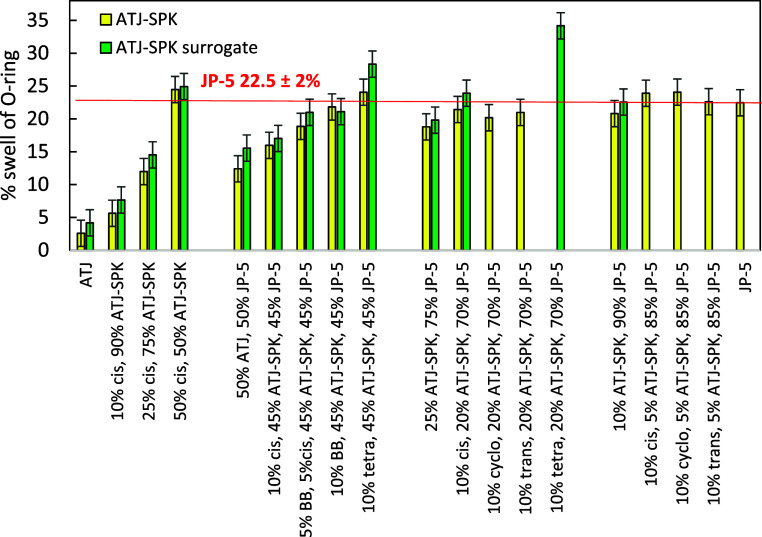
Swelling of AS Buna-N O-rings upon exposure to ATJ-SPK or ATJ-SPK surrogate mixtures containing *cis*-decalin (cis), butylbenzene (BB), tetralin (tetra), *trans*-decalin (trans), and cyclohexane (cyclo). The percentages in the *x*-axis caption are all vol % of each component in the mixture.

All dopants (*cis*-decalin, *trans*-decalin, cyclohexane, and tetralin) when mixed at 10 vol % in 20 vol % ATJ-SPK and 70 vol % JP-5 mixtures elevated the swelling of the AS Buna-N O-ring to that found for JP-5 within the error of the measurement. The dilution of JP-5 (35.6% cycloalkanes, 10.2% alkylbenzenes and diaromatic compounds, and 5.4% cycloaromatic compounds) with ATJ-SPK lowers the concentration of the components that enhance swelling. The addition of 10 vol % of dopants does not return these component concentrations to their original levels, but they do bring the swelling values to within the error found for JP-5. This result is not unexpected. First, these components may have more favorable interactions with the polymer. Second, the dopants fall on the smaller size end of the range of compounds found in the fuel (see complete compositional analysis of the JP-5 in the Supporting Information). For ethyl-, propyl-, and butylbenzene, researchers have shown that the smaller compounds induced greater swelling when mixed at low concentrations with synthetic fuels,^2,7^ so it is possible for these dopants to swell more than the original components, thus less is needed to reach the same level of swelling. For the mixtures of 45% ATJ-SPK and 45% JP-5, only the aromatic additives were able to raise the level of swelling back to that induced by JP-5.

The SLA acrylate O-rings exhibited similar trends for mixtures of ATJ-SPK with JP-5, but the overall amount of swelling was smaller (Figure 8). The JP-5 mixtures containing 10% ATJ-SPK produced swells that were within the error of the measurement for JP-5 (18 ± 2% swell). Increasing the amount of ATJ-SPK in the mixtures lowered the swelling significantly. The SLA acrylate O-ring swellings were 15.5% and 12.5% when exposed to 25 vol % ATJ-SPK in JP-5 and 50 vol % ATJ-SPK in JP-5, respectively. To increase the swelling of 50 vol % ATJ-SPK in JP-5, dopants were added. Adding 10% *cis*-decalin to a 50/50 mixture had little effect on the swelling, while doping with 10% tetralin had a large effect, raising the swelling to higher levels than that of JP-5 itself.

**Figure 8:**
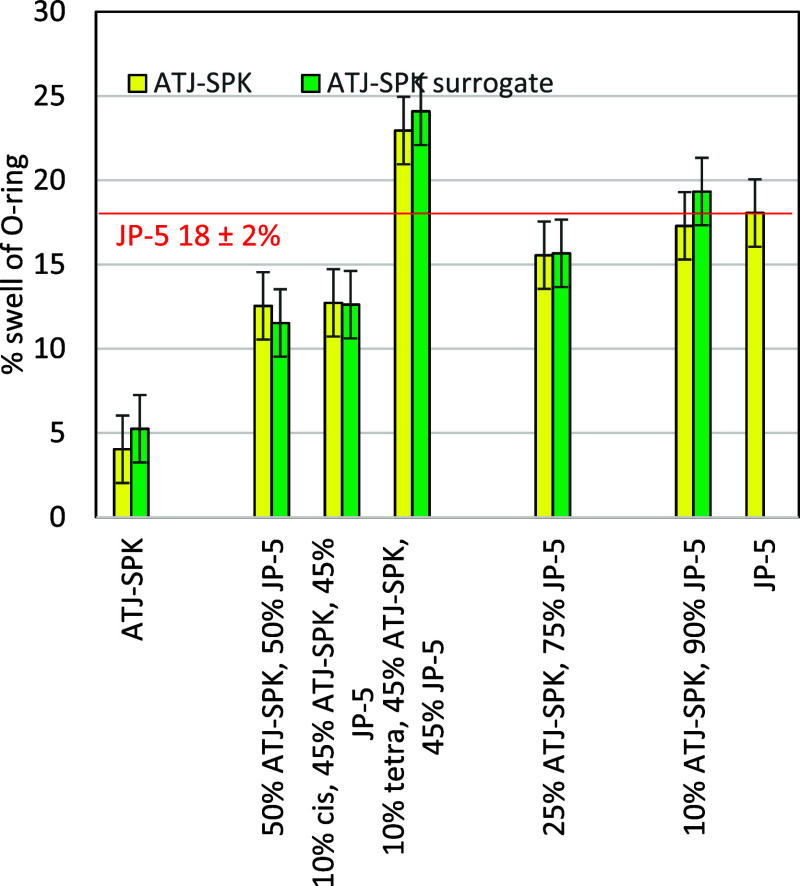
Swelling of SLA O-rings upon exposure to ATJ-SPK or ATJ-SPK surrogate mixtures containing *cis*-decalin (cis) and tetralin (tetra). The percentages in the *x*-axis caption are all vol % of each component in the mixture.

Another goal of the study was to determine the amount of swelling of the O-rings upon exposure to the ATJ-SPK surrogate. Figure 7 also shows that the swellings of AS Buna-N after exposure to mixtures made with the surrogate were slightly higher than those found for ATJ-SPK but within error of the measurements. For the SLA acrylate O-rings, the swellings induced by ATJ-SPK mixtures were very close to the swellings produced by the surrogate (Figure 8). The exact compounds in ATJ-SPK and its surrogate will differ slightly, which could cause small differences in swelling. It is possible that the isomers in ATJ-SPK are more highly branched and may penetrate the Buna-N less.

### 3.3 Tensile Strength Experiments

The tensile strength of the SLA acrylate O-rings that had not been exposed to the fuels and mixtures was smaller (6 ± 1 MPa) than that of the AS Buna-N O-ring (10 ± 1 MPa). These values are consistent with previous studies using different lots of SLA (4.6 ± 0.6 MPa) and AS Buna-N (11 ± 1 MPa) O-rings.^14^ The print orientation for the SLA polymer was slightly different in the current study, specifically with the number of supports in the current study being greater than that in previous studies. The design for the additive manufacturing (DFAM) aspect of the SLA O-rings such as print orientation, raft size, supports (number, location, touchpoint size, etc.), peel forces, postprocessing, and support removal did not appear to affect the geometry or tensile properties.

Both SLA and AS Buna-N O-rings lost tensile strength as they swelled from the uptake of fuels and organic components. Exposure of AS Buna-N O-rings to jet fuel for a week caused a 32% reduction in tensile strength (Figure 9A). The greatest loss in tensile strength (40%) occurred for the O-ring that swelled the most (34% swell, 10% tetralin, 20% ATJ-SPK surrogate, 70% JP-5). Exposure of SLA O-rings to JP-5 jet fuel for a week caused a 43% reduction in tensile strength (Figure 9B). In general, for both O-rings, as the swelling increased, the tensile strength decreased, as shown in Figure 9C. A comparison of mixtures with ATJ-SPK and the ATJ-SPK surrogate shows that most of their tensile strengths are consistent with this trend. The slightly higher Buna-N O-ring swells found for the ATJ-SPK surrogates resulted in lower tensile strengths. The uptake of organic compounds loosened the interaction of polymer chains, allowing them to be more easily broken.

**Figure 9:**
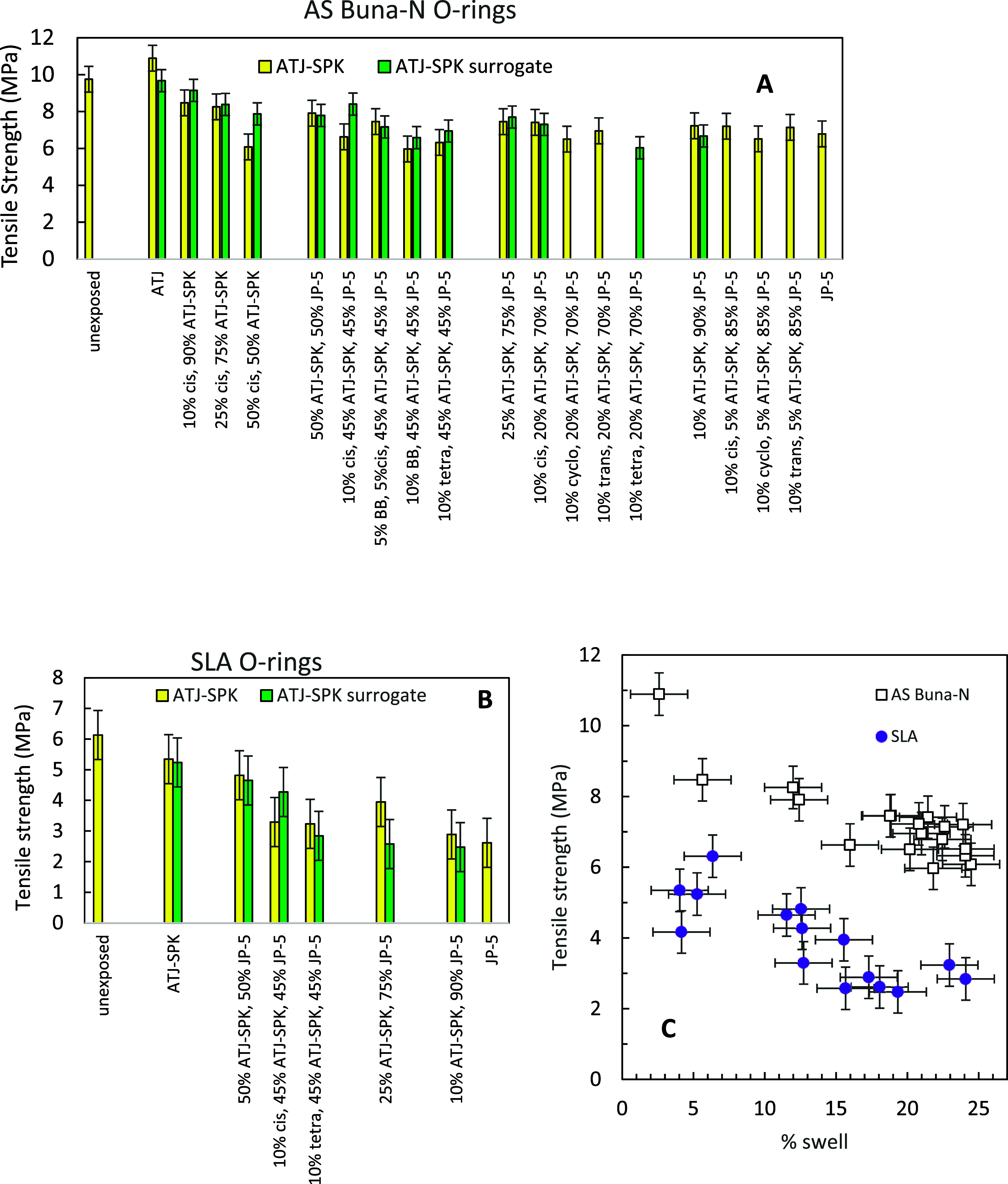
Tensile strength of O-rings before and after exposure to fuels and mixtures: (A) AS Buna-N O-rings and (B) SLA O-rings. (C) Loss of tensile strength due to the swelling of the polymers. Additives include *cis*-decalin (cis), butylbenzene (BB), tetralin (tetra), *trans*-decalin (trans), and cyclohexane (cyclo). The percentages in the *x*-axis caption are all vol % of each component in the mixture.

The first aspect of this work sought to determine the physical properties of the JP-5, ATJ-SPK, and ATJ-SPK surrogates and mixtures with various dopants. This study differs from other studies that have focused on commercial aviation turbine fuel, which differs from JP-5 with its lower flash point (38 °C) and differing range of densities (775 to 840 kg·m^3^).^42^ With the 60 °C minimum flash point for JP-5, two groups of mixtures met the specification: (1) mixtures with 5% ATJ-SPK, 85% JP-5, and additives with higher flash points (not cyclohexane) and (2) mixtures with 20% ATJ-SPK, 70% JP-5, and dopants with higher flash points. The flash points of the dopants used were −18 °C (cyclohexane, bottle label from Alfa Aesar), 55 °C (*trans*-decalin), 62 °C (*cis*-decalin), and 76 °C (tetralin). When there are efforts to enhance O-ring swelling by using additives, flash points need to be considered. When examining the flash points of *n*-alkylcyclohexanes and *n*-alkylbenzenes, compounds larger than *n*-pentylcyclohexane (63 °C, Fisher Scientific Safety data sheet) and *n*-pentylbenzene (65.3 °C)^45^ have flash points higher than the military specification, but these longer chain compounds tend to be expensive and may swell less due to their larger size. Bicyclohexyl, which has a flash point of 92 °C (Fisher Scientific Safety data sheet), is more reasonably priced but still more expensive than butylcyclohexane (flash point of 48 °C).^45^ The *cis*-decalin and tetralin explored herein are better candidates for swelling enhancement as well as flash point.

Another aspect to be considered is the combustion of mixtures of ATJ-SPK and JP-5. No work has been reported for ATJ-SPK/JP-5 combustion in jet engines. Work in the author’s laboratory, however, has shown that mixtures of 40% ATJ-SPK and 60% JP-5 did not combust well in a diesel engine with the engine not reaching its rated speed within 30 s for cold engine starting.^46^ The military will use jet fuel in diesel engines under certain circumstances. Diesel engine combustion can be assessed by looking at the cetane number. Military diesel fuel must have a cetane number of 42 or higher. A reported cetane value of ATJ-SPK is 18, while a 30% ATJ-SPK in JP-5 mixture had a cetane value of 40.4.^41,46^ Dopants can raise or lower the cetane number. The cetane numbers of the dopants used in the current study are 42 (*cis*-decalin), 32 (*trans*-decalin), 19–20 (cyclohexane), 12–13 (butylbenzene), and 9–21 (tetralin).^47^ In general, aromatic compounds have low cetane numbers, which adversely impact diesel engine combustion. Further work is needed to explore how dopants that enhance swelling change the cetane numbers of ATJ-SPK/JP-5 mixtures.

The blending explored in this study can be placed in the context of ASTM D7566, the Standard Specification for Aviation Turbine Fuel Containing Synthesized Hydrocarbons.^48^ In section 6.1.5, it states, “Conventional blending components or Jet A or Jet A-1 fuel certified to Specification D1655; with up to 50% by volume of the synthetic blending component defined in Annex A5.”^48^ Annex A5 covers ATJ-SPK from ethanol and iso-butanol and states, “A5.4.1 ATJ-SPK synthetic blending components shall be comprised of hydro-processed synthesized paraffinic kerosene wholly derived from ethanol^21^ or iso-butanol^22^ (see Note A5.1) processed through dehydration, oligomerization, hydrogenation, and fractionation”.^23^ An earlier review reported a lower limit for ATJ-SPK from iso-butanol, with its maximum blending fraction being 30% ATJ-SPK in commercial aviation fuel.^49^ That ATJ-SPK consisted of iso-alkanes of 8, 12, or 16 carbons when starting from iso-butanol.^49^ The current study explores the blending of JP-5 and ATJ-SPK with up to 50% ATJ-SPK. These 50/50 mixtures required aromatic compounds to raise the level of swelling to that of JP-5. Mixtures with 25% ATJ-SPK in JP-5 produced O-ring swelling with levels similar to that of JP-5, suggesting that this blending ratio would have minimal impact on swelling without the addition of dopants. It is important to note that currently, the military specification MIL-DTL-5624X has only approved the use of ATJ-SPK derived from ethanol.

This specification also requires that blends of synthesized fuel with Jet A have between 8 and 25% aromatic content.^48^ The JP-5 used in this study contains 15.6% aromatic components [10.2% alkylbenzenes and diaromatic compounds and 5.4% cycloaromatic compounds (like tetralin)]. The mixtures in the current study that have an aromatic content between 8 and 25% are (1) all mixtures containing 70% or more JP-5 and (2) mixtures containing 45% JP-5 and 5% or more added aromatic dopant. These mixtures with ATJ-SPK induced AS Buna-N O-ring swells between 18.9 and 24.1%, which is close to the value for JP-5 of 22.5 ± 2%. The AS Buna-N O-ring tensile strength after exposure to these mixtures ranged from 6.3 to 7.5 MPa, which falls within the error of 6.8 ± 0.7 MPa for JP-5. The solution of 10% *cis*-decalin with 45% JP-5 and 45% ATJ-SPK does not have a high enough aromatic content and its swell is only 16%.^12^

The size of the aromatic compounds can impact swelling. Romanczyk et al.^7^ doped Sasol IPK with 8% aromatic compounds and found the trend in induced Buna-N swelling to be ethylbenzene (3.1%) > propylbenzene (1.9%) > butylbenzene (1.4%).^7^ The Buna-N swelling trend reported by Graham et al.^11^ for doping synthetic fuel S-5 (FT-SPK) with 10% aromatic compounds was toluene (8.94%) > ethylbenzene (8.67%) > propylbenzene (7.96%) > pentylbenzene (5.75%). The trend was less clear in work by Faulhaber et al.^2^ who doped ATJ-SPK with 8% of various compounds. For the *n*-alkylbenzenes tested at 22 °C, the order of Buna-N swelling was propylbenzene (4.9% swell) > butylbenzene (3.9% swell) > heptylbenzene (3.1% swell), but the swell from hexylbenzene was 4.3%. If the swelling differs based on size, then it might be expected that Jet A, which contains smaller compounds with lower flash points, would swell more than JP-5. Faulhaber et al.^2^ report Buna-N swells of 11.9%, 13.2%, and 13.6% for JP-8 (9.5% aromatic content), Jet A (16.4% aromatic content), and JP-5 (18.4% aromatic content), respectively. If the aromatic size had no effect, we might expect a linear increase in swelling with aromatic content. The trend of data reported for these three fuels is not linear, which suggests that the size does have an effect. The differences in overall swell, however, are small, which may mean that swelling information from one fuel could be used to provide a reasonable estimate of other fuels when their aromatic contents are the same.

In addition, when comparing the swelling of behavior of short-chain alkylbenzenes discussed above, the interaction of the polymers with the fuel components can be explored using Hansen Solubility Parameters (HSPs), which separate interactions into dispersion (δ_D_), polarity (δ_P_), and hydrogen bonding (δ_H_).^50^ The difference between the HSPs of a polymer and the interacting organic component (eq 3) can be calculated and is designated as “R_a_”, and this can be compared with that of the “radius”, *R*_o_, for the specific polymer.^50^ If the ratio of *R*_a_/*R*_o_ (called the relative energy distance, RED) is less than one, then the solvent and solute are “compatible”, which would indicate higher swelling. The δ_Dp_, δ_Pb_, δ_Hp_, and R_o_ values for Buna-N have been reported to be 17.8, 3.2, 3.4, and 3.7, respectively.^50^

3 $$R_{a}=\sqrt{4\times {\left(\delta_{Dp}-\delta_{Ds}\right)}^{2}+{\left(\delta_{Pp}-\delta_{Ps}\right)}^{2}+{\left(\delta_{Hp}-\delta_{Hs}\right)}^{2}}$$

In eq 3, the HSPs for the polymer are δ_Dp_, δ_Pb_, and δ_Hp_ and for the organic solvent are δ_Ds_, δ_Ps_, and δ_Hs_. The values for Buna-A can be used with the δ_Ds_, δ_Ps_, and δ_Hs_ for toluene (18, 1.4, and 2), ethylbenzene (17.8, 0.6, and 1.4), propylbenzene (17.3, 2.2, and 2.3), and butylbenzene (17.4, 0.1, and 1.1) to calculate *R*_a_ values of 2.32, 3.28, 1.79, and 3.94 for these compounds, respectively.^50^ The resulting RED values are 0.63, 0.89, 0.48, and 1.07 for toluene, ethylbenzene, propylbenzene, and butylbenzene, respectively. These values suggest that toluene, ethylbenzene, and propylbenzene are good solvents (RED < 1), which is consistent with the data discussed in the previous paragraph. When the polymer and solvent have similar dispersion, polarity, and hydrogen bonding HSPs, then swelling should improve. Studies have shown that changing the formulation of Buna-N by creating composites with materials such as polyamide can alter the swelling of the polymers.^51^ The smallest RED value of 0.48 suggests that propylbenzene should induce the most swelling, which was not seen in past or current studies, which further suggests that size may be a factor in swelling.

The Buna-N O-rings in the current study exhibit greater swelling than those used by Faulhaber et al.^2^ The ATJ-SPK swelled their Buna-N O-rings by 0.5%, while the O-rings in the current study swelled by 3%. Based on their kinetic plots, at 168 h (7 days), the value appears to be close to their reported equilibrium value of 0.5%. Also, their higher aromatic content JP-5 (18% aromatic) produced a lower swelling of 13.6% at a higher temperature (37 °C) than the 22.5% swelling found in the current study for a lower aromatic content JP-5 (10.2% alkylbenzenes and diaromatic compounds and 5.4% cycloaromatic compounds) at 21 °C. Finally, the slightly lower 8 wt % *cis*-decalin in ATJ-SPK yielded a 1.7% swell, which is lower than the 5.6% swell found for the 10 wt % *cis*-decalin in ATJ-SPK in the current study. The variations in the two studies may be caused by differences in (1) the lot of ATJ-SPK tested, (2) specific distribution of aromatic and cyclic compounds in the fuel, (3) lot and type of Buna-N O-rings used, or (4) form of the O-ring tested (slices of O-ring versus whole O-ring). As discussed earlier in this work, the MS Buna-N O-rings swelled much less than the AS Buna-N O-rings, so the specific Buna-N O-ring used is important. While the specific values of swelling differed, the general trends found were the same. The compounds with aromatic functional groups enhanced the swelling of Buna-N O-rings more than did cyclic compounds.

The formulation of the ATJ-SPK surrogate was successful. The percentage difference between the surrogate’s and ATJ-SPK’s properties was 4%, 0.04%, 0.03% and 0.2% for viscosity, density, speed of sound, and flash point, respectively (Table 1). The swelling behavior of both the SLA and Buna-N O-rings showed only small differences when the surrogate was used in place of ATJ-SPK. The physical properties of the mixtures containing the surrogate were also close to those made from ATJ-SPK. The surrogate could be improved by using higher-purity *iso*-dodecane, such as using 2,2,4,6,6-pentamethylheptane. The trade-off with using this isomer is that it is more expensive.

The methacrylate SLA O-rings used in this study exhibited less swelling (18%) than did the Buna-N O-rings (22.5%) when exposed to the jet fuel. Parker Hannifin states that O-ring swelling should be below 30% for static applications, and less than 10% for dynamic applications, but shrinkage should be avoided to prevent leakage.^52^ The ability of the SLA O-rings to swell to a reasonable level suggests that they could be used in an emergency in a diesel engine. The current 3D printing process for the O-rings used in this study produces surface features (indents) that may be too large to pass normal O-ring production standards, but they may work well if needed in an emergency.

This work sought to formulate a surrogate for ATJ-SPK, to determine the impact of mixtures of JP-5 with ATJ-SPK and its surrogate with and without dopants on the swelling and tensile strength behavior of commercial Buna-N and SLA O-rings and to explore the effect of mixing ATJ-SPK with JP-5 on fuel physical properties. In general, the surrogate was successfully formulated, and its properties and ability to induce O-ring swelling were similar to those of ATJ-SPK. Of the additives tested at 10 wt %, the ones with aromatic functionality were able to raise the swelling of mixtures with 45% ATJ-SPK and 45% JP-5 to the same swelling as JP-5. The cyclic compounds enhanced swelling to a smaller degree. It is important to emphasize that this study explores the use of O-rings for diesel engine applications and not jet engine applications. Extension of this work to jet engines would require further testing. Finally, the ability to additively manufacture methacrylate O-rings without requiring precise and tightly controlled DFAM parameters facilitates expedited printing of SLA O-rings. The reasonable swelling behavior of the SLA O-rings suggests that they could be quickly implemented in an emergency. Future work is recommended to investigate SLA systems with other curing methods such as digital light projectors to further decrease manufacturing time and research investigating other AM methods that utilize thermoplastics, such as fused deposition modeling and selective laser sintering.

## 4 Conclusions

In this work, the swelling and tensile strength behavior of Buna-N and additively manufactured acrylate O-rings were explored after exposure to mixtures containing synthetic ATJ-SPK, its surrogate, JP-5, and additives containing cyclic and aromatic functional groups. The aromatic additives enhanced the swelling more than the cyclic compounds and enabled mixtures with 45% ATJ-SPK and 45% JP-5 to produce the same level of swelling of Buna-N O-rings as that induced by JP-5. This work also successfully formulated a surrogate for ATJ-SPK based on matching the density, viscosity, speed of sound, and flash point. The surrogate produced swelling behavior and mixture properties similar to those of the ATJ-SPK. Measured physical properties indicate that many of the dopant systems were able to meet the density requirements of JP-5, while fewer of them were able to meet the flash point minimum. The *cis*-decalin and tetralin were dopants that had flash points that are higher than the military minimum. For ATJ-SPK/JP-5 mixtures to be used successfully in diesel engines, the cetane number of dopants must be considered. Finally, the ability of the SLA O-rings to swell to a reasonable level suggests that they may currently be a viable option for contingency operations in a diesel engine and that future AM O-rings may be viable for regular use.

## Supplementary Material



## Notes

Mass percentage of tier 2 compounds found in JP-5 by functional group (or functionality) and number of carbons using GC × GC/FID, swelling measurements of MS Buna-N and Ford O-rings with ATJ-SPK and ATJ-SPKs (surrogate) at intermediate times and on the seventh day, swelling measurements of AS Buna-N O-rings with ATJ-SPK mixtures at intermediate times and on the seventh day, swelling measurements of AS Buna-N O-rings with ATJ-SPKs (surrogate) mixtures at intermediate times and on the seventh day, and swelling measurements of additively manufactured SLA O-rings with ATJ and ATJs (surrogate) mixtures at intermediate times and on the seventh day (PDF)
