# Supplementary material for: Determining the Swelling Behavior and Tensile Strengths of Commercially Produced Buna‑N O‑Rings and Stereolithographic Additively Manufactured O‑Rings after Exposure to Mixtures Containing Jet Fuels, Synthetic Fuels, and Fuel Surrogate
Source: ACS Omega. 2025 Jul 24;10(30):33777–87. doi: 10.1021/acsomega.5c04803 (PMC12332619; doi:10.1021/acsomega.5c04803)
Supplement: Supplementary file 1 [file ao5c04803_si_001.pdf]

Supporting information

Determining the swelling behavior and tensile strengths of commercially-produced Buna-N O-rings and stereolithographic additively manufactured O-rings after exposure to mixtures containing jet fuels, synthetic fuels, and fuel surrogate

Dianne J. Luning Prak[a,*]
David Graham[a]
Kara Hunt[a]
Micah Evans[a]
Terrence Dickerson[b]
Jonathan Slager[c]
Jim S. Cowart[c]

(a) Chemistry Department U. S. Naval Academy, 572M Holloway Road, Annapolis, MD 21402, United States

(b) Naval Air Warfare Center Aircraft Division (NAWCAD) Air Systems Group; Building 2360, NAS Patuxent River, MD 20670, United States

(c) Mechanical and Nuclear Engineering Department U. S. Naval Academy, 590 Holloway Road, Annapolis MD, 21402, United States

*Email: prak@usna.edu

The isododecane isomers were analyzed using gas chromatography/mass spectrometry. The sample was dissolved in dichoromethane and analyzed using method given in Luning Prak et al.[1] Based on peak area, the major components were 90% 2,2,4,6,6-pentamethylheptane, 7.5% 2,2,4,4-tetramethyloctane, 1.7% 2,2,3,3-tetramethylpentane, 0.8% 2,5,9-trimethyldecane. The isododecanes are 98% of mixture, which is larger than specified on the bottle.

The chemical composition of the JP-5 is given in Table S1.

Table S1. Mass Percentage of Tier 2 Compounds Found in JP-5 by Functional Group (or Functionality) and Number of Carbons Using GC×GC/FID

carbon number	*n*-alkanes average mass %	iso-alkanes average mass %	mono-cyclo-alkanes average mass%	di-cyclo-alkanes, average mass %	tri-cyclo-alkanes, average mass %	alkyl-benzene compounds average mass %	cyclo-aromatics, average mass %	di-aromatic compound, average mass %
**C8**	0.1	0.1	0.2			0.1		
**C9**	0.3	0.3	0.6	0.1		0.8		
**C10**	1.6	1.3	2.2	1.0		2.0	0.5	
**C11**	5.2	5.7	5.3	2.9	0.1	2.7	1.9	0.3
**C12**	5.4	6.7	7.3	3.0	0.1	1.9	1.7	0.4
**C13**	3.8	6.7	5.2	2.3		1.4	1.0	0.2
**C14**	1.9	4.6	2.9	0.9		0.4	0.3	
**C15**	0.8	2.7	1.3	0.1				
**C16**	0.2	1.0	0.1					
**C17**	0.1	0.2						
Total	19.4	29.3	25.1	10.3	0.2	9.3	5.4	0.9

The plots showing the approach to equilibrium for the O-rings systems are shown in Figures 1-4.

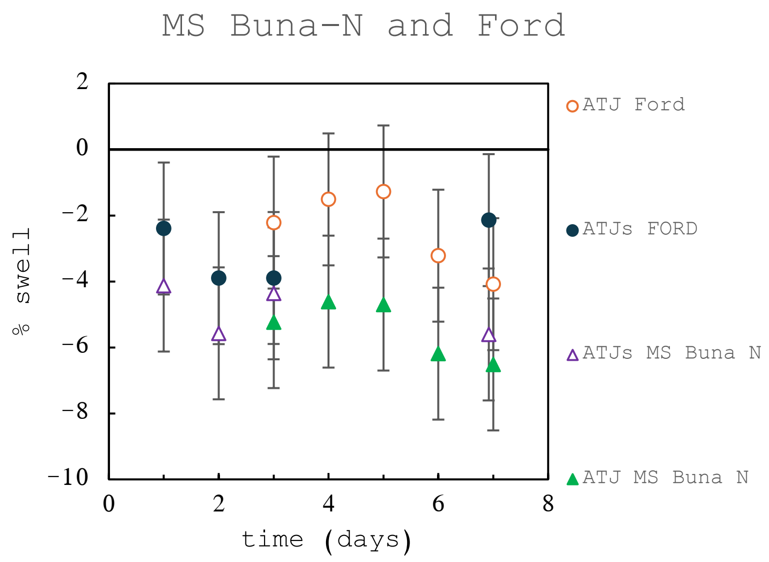

Figure S1. Swelling measurements of MS Buna-N and Ford O-rings with ATJ-SPK and ATJ-SPKs (surrogate) at intermediate times and on seventh day. (To save space, the word "SPK" is not on the plot.)

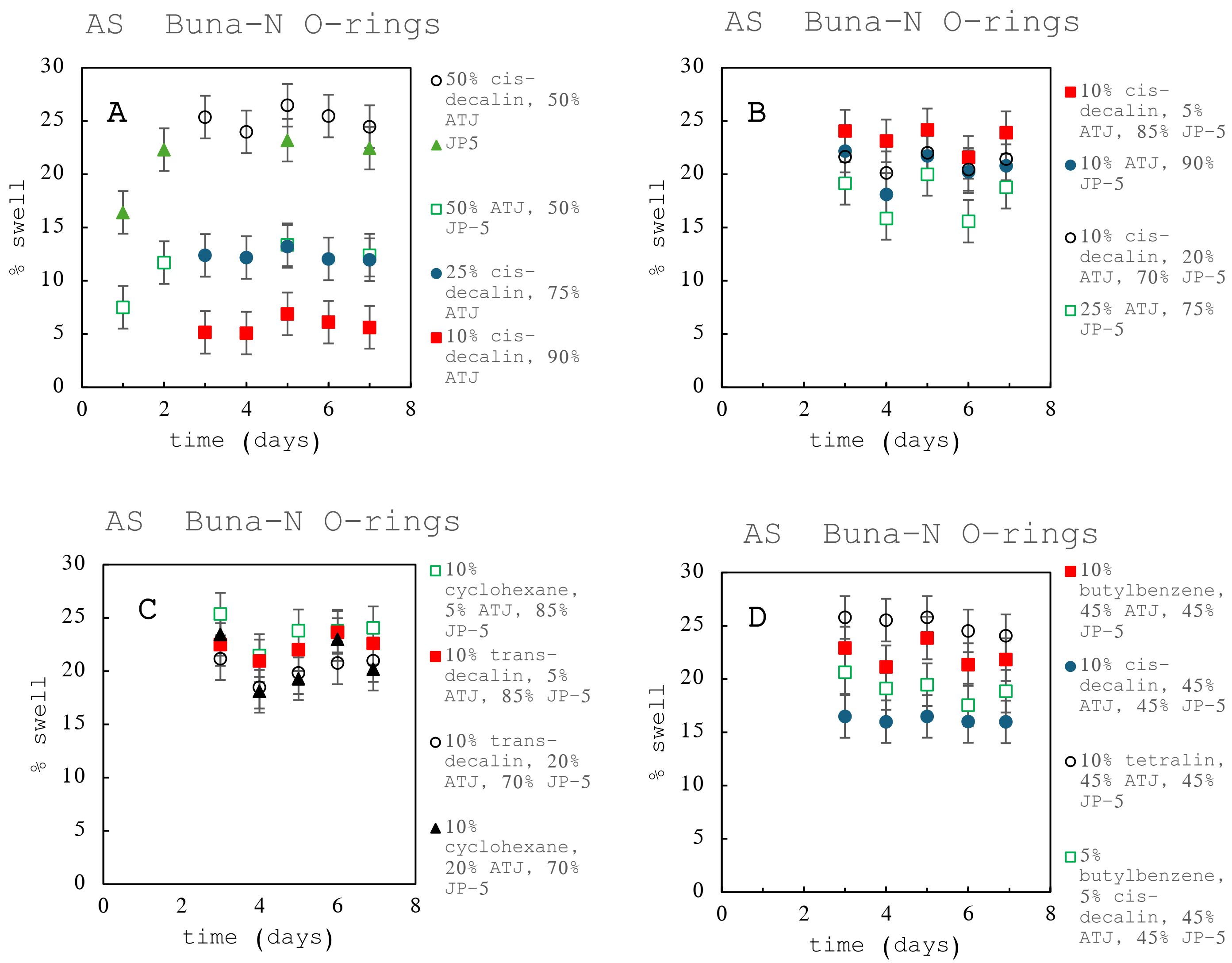

Figure S2. Swelling measurements of AS Buna-N O-rings with ATJ-SPK mixtures at intermediate times and on seventh day. (To save space, the word "SPK" is not on the plot.)

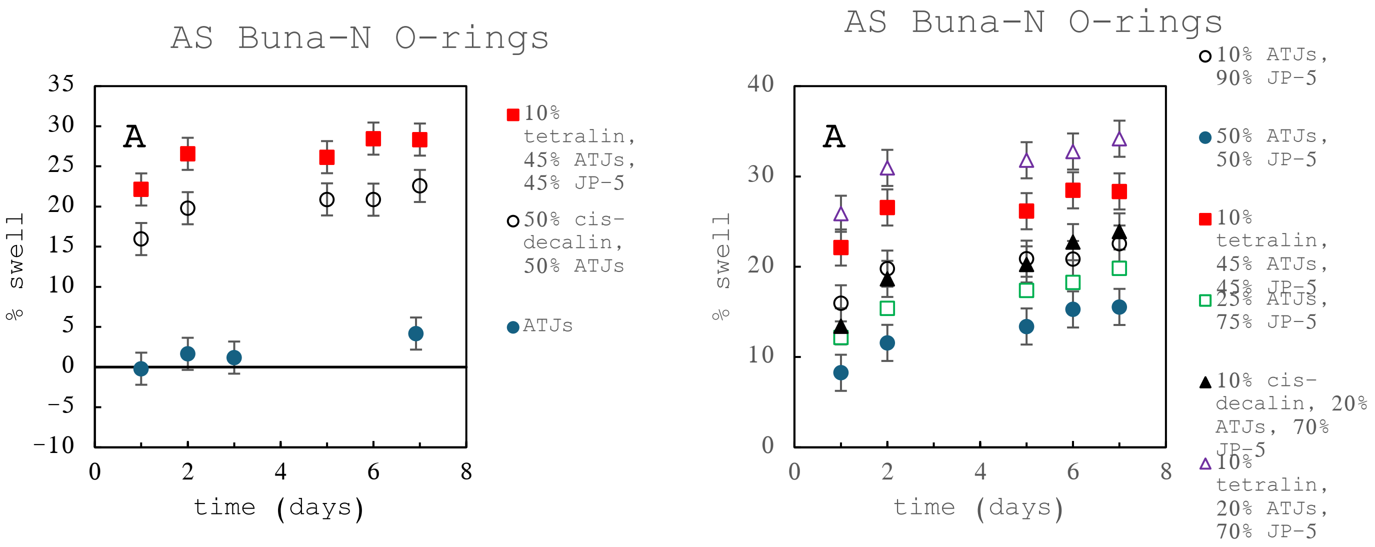

Figure S3. Swelling measurements of AS Buna-N O-rings with ATJ-SPKs (surrogate) mixtures at intermediate times and on seventh day. (To save space, the word "SPK" is not on the plot.)

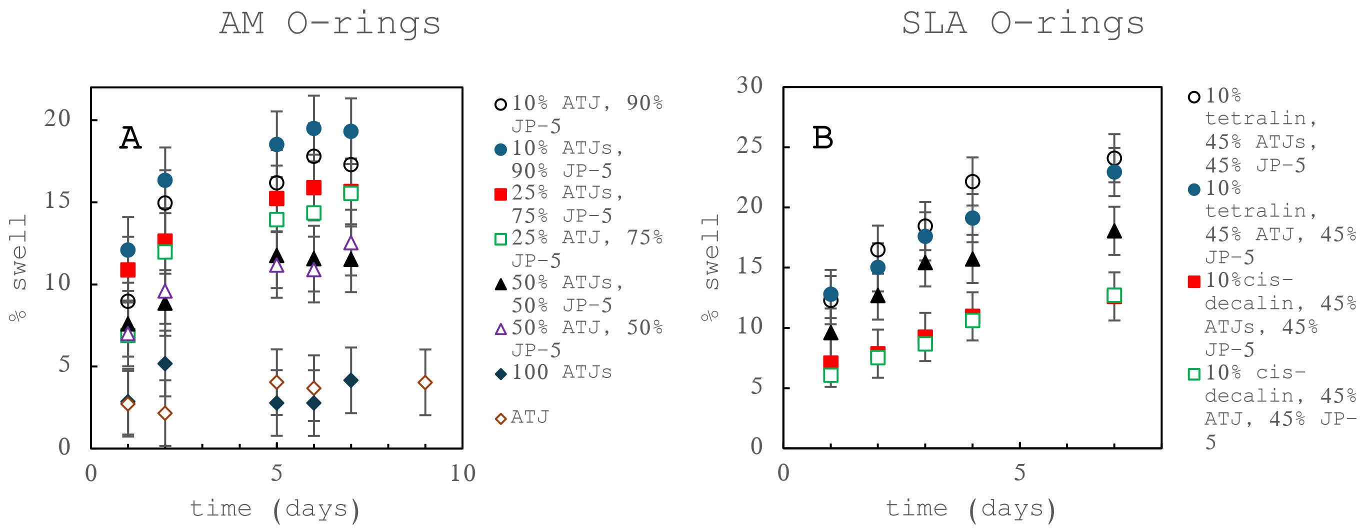

Figure S4. Swelling measurements of additively-manufactured SLA O-rings with ATJ and ATJs (surrogate) mixtures at intermediate times and on seventh day. (To save space, the SPK is not on the plot.)

References

1. Luning Prak, D. J.; Luning Prak, P. J.; Trulove, P. C.; Cowart, J. S. Formulation of surrogate fuel mixtures based on physical and chemical analysis of hydrodepolymerized cellulosic diesel fuel. *Energy Fuels* **2016**, *30*, 7331-7341. 10.1021/acs.energyfuels.6b01114.
